# A pathway towards the development and evolution of consumer behavior: Policy directions for sustainable development and improvement of nutrition

**DOI:** 10.3389/fnut.2022.1066444

**Published:** 2022-12-02

**Authors:** Fang Su, Jiangbo Chang, Xing Zhang, Shah Fahad, Shimza Bint Aslam

**Affiliations:** ^1^School of Economics and Management, Northwest University, Xi’an, China; ^2^School of Economics and Management, Shaanxi University of Science and Technology, Xi’an, China; ^3^School of Economics and Management, Zhengzhou University of Light Industry, Zhengzhou, China; ^4^School of Economics and Management, Leshan Normal University, Leshan, China; ^5^School of Management, Hainan University, Haikou, China; ^6^Institute of Agricultural and Resource Economics, University of Agriculture, Faisalabad, Pakistan

**Keywords:** sustainable food production, formation mechanism, influencing factors, evolutionary game, online food trading market, lemon problem

## Abstract

**Introduction:**

The virtuality, concealment, uncertainty and complexity of online trading make the online food trading market have security risks, while the lack of information, information asymmetry and imperfect market system make the “lemon problem” in the market increasingly obvious.

**Methods:**

In order to clearly understand and manage the “lemon problem” in the online food trading market, we built an evolutionary game model involving the seller, buyers and online food trading platform, deeply analyzed the formation process of the “lemon problem” in the online food trading market, and revealed the influencing factors and effects of each subject’s strategy choice from the perspectives of subsidy, punishment, cost, and benefit.

**Results:**

Findings of this study reveal that: (1) In the online food trading market, the strategy of the seller, buyer and platform will be stable in six situations, and the “lemon problem” will emerge with the development and evolution of the online food trading market. (2) The strategy of each subject in the online food trading market will be affected by variables like cost difference between positive performance and negative performance of the seller, punishment from the buyer with positive participation to the seller with negative performance, subsidy from the platform with positive regulation to the seller with positive performance, etc., and different factors have different influence directions and degrees on the subject strategy. (3) In the online food trading market, cost, punishment, subsidy and benefit have different effects on the subject’s strategy. Among them, cost and cost difference have the most significant impact on the subject’s strategy, followed by punishment and benefit difference, and subsidy and additional benefit have less impact on the subject’s strategy.

**Discussion:**

Based on our study findings, it is proposed that by constructing a complete and standardized system of online food trading market from the aspects of examination and verification institution, reward and punishment institution, and supervision institution, it will be able to provide reference for managing the “lemon problem” in the online food trading market, promoting the sustainable development of the market, and ensuring the safety of online food.

## Introduction

In recent years, driven by the upsurge of “Internet +” and “platform economy,” the food supply chain closely related to consumer life has been constantly changing, and the marketing mode represented by Internet platform sales has gradually emerged. At present, people’s lives are full of various online food trading platforms, such as Meituan Takeout, Eleme, Koubei, and Jingdong To Home from China, Grubhub, Ubers Eats, and DoorDash from United States, Deliveroo and Just Eat from United Kingdom, Delivery Hero from Germany, and Swiggy from India (1). During the COVID-19 pandemic, in order to reduce the spread of the virus, some countries adopted partial or complete blockade measures, such as closing schools, workplaces, entertainment places, restaurants, etc. (2), which had a significant impact on people’s food purchasing methods and consumption habits (3), and changed consumers’ dietary preferences from offline eating to online delivery (4). In this process, people’s demand for online food services such as takeout catering and fresh food e-commerce is also expanding. Globally, online food trading market revenue increased by 27% in 2020, reaching 136.4 billion dollars. Furthermore, a 79% increase in total orders between 2020 and 2021, across its 17 operating countries including UK, Germany, Canada, and Netherlands (5). In 2021, China’s total income in food delivery is 27.3 billion dollars, and the United States’ total income is 15 billion dollars. The China Sharing Economy Development Report (2022) shows that in 2021, online takeout revenue will account for 21.4% of China’s catering industry revenue, up 4.5% year on year (6). According to the Research on NPS User Experience of Fresh Food in 2021 by iiMedia Research, the scale of China’s fresh food e-commerce industry in 2021 will be 458.5 billion yuan, an increase of 46.2% over 2020 (7).

Online food trading is a new economic model based on emerging information technologies such as the Internet, big data, artificial intelligence, and blockchain, which has different characteristics and patterns from traditional food trading. While expanding the market scale and creating social and economic benefits, the “lemon problem”^1^ also comes into being (8–11). The virtuality, concealment, uncertainty, and complexity of online trading make it increasingly obvious that food is not fresh, delivery is not timely, after-sales service is poor, false promotion, and other issues (12), which has aroused widespread concern of the government and society. In order to meet the new challenges of online food security, countries have taken a series of response measures. Among them, German consumers have a series of rights and interests protection policies, such as the right of inspection (unsatisfied food users can return goods on the spot). The United States government has established a special food safety website to uniformly and authoritatively disclose food safety information. Japan’s Food Hygiene Law clearly stipulates online food, and food safety is subject to the double strict supervision of law and public opinion. China’s food safety regulatory authorities have successively issued such rules and regulations as the Food Safety Law, the Measures for the Investigation and Punishment of Illegal Acts of Online Food Safety, and the Measures for the Supervision and Administration of Food Safety in Online Catering Services, which clearly stipulate the food safety responsibilities and obligations of online food trading platforms, food producers, and food operators (13, 14). However, the problem of online food safety has not been fundamentally solved. The reality of many businesses, difficult supervision, limited number of technical and law enforcement personnel makes it difficult to solve the “lemon problem” in the online food trading market. In 2020, China’s 12315 platform, an Internet platform dedicated to handling consumer complaints, received 65,800 online food complaints (7). For a long time, consumers will lose confidence in the market due to their inability to distinguish the quality of food. High quality businesses will be difficult to obtain income matching the quality of food due to consumers’ distrust. Food trading platforms will face difficulties such as low customer retention rate, high churn rate, and low profits. Eventually, the online food trading market will be full of low-quality businesses, and food quality also has high safety risks (15).

Online food trading involves government, food suppliers, food consumers, food trading platforms, media, and other subjects, as well as production, transportation, sales, after-sales, and other links. In the transaction process, many factors, such as cost and income, will affect the strategic choice behavior of each subject. In relevant research, scholars believe that the lack of information, information asymmetry and imperfect market system will lead to the “lemon problem” in the online food trading market (16), and the “lemon problem” in the market can be alleviated with the help of blockchain technology, reputation mechanism, and regulatory mechanism (17, 18). However, will the “lemon problem” in the online food market definitely exist? What factors will affect the “lemon problem?” What is the relationship between the behavior of each subject in the market and the “lemon problem?” How to alleviate and manage the “lemon problem” in the online food trading market? We do not know these. To effectively solve the lemon problem in the online food trading market, it is necessary to analyze the formation mechanism of formation of the “lemon problem” and clarify the key factors affecting the development and evolution of the online food trading market. Only by recognizing and solving problems can the interests and needs of food suppliers, food consumers, and other subjects be met, and the online food trading market can achieve long-term, healthy and sustainable development.

Under the above background, we analyzed the online food trading market and built an evolutionary game model with the seller, buyer and platform as the main body. By depicting the interaction between the subjects in the online food trading market and revealing the influencing factors and effects of each subject’s strategy choices, we hope to provide reference for managing the “lemon problem” in the online food trading market, promoting the sustainable development of the market, and ensuring the online food safety.

## Literature review

Food safety concerns the health of the people and the long-term stability of society (19). With the continuous optimization and improvement of network information technology and network infrastructure, Internet thinking and network development mode have permeated all areas of social life, and the online food market has developed rapidly. The online food trading has changed the consumption mode, trading mechanism and circulation link of traditional food, and has also increased the difficulty of food safety management while facilitating consumers. In the relevant research on online food safety, scholars mainly focus on the causes and governance of online food safety problems.

On the one hand, the causes of online food safety problems. With the continuous expansion of the scale of online food trading market, the market has shown new features such as prominent platform effect (forming a new economic development model with platform organization and its data control right as the core), data oriented consumption (individual consumption behavior is affected by data information), and increased information asymmetry (information asymmetry mastered by various subjects) (12). In this process, problems such as weak performance of platform responsibilities, imperfect market credit evaluation system, lagging government regulatory capacity, and acute contradiction between the platform and the seller have become increasingly prominent (11, 20). The characteristics of online food trading, such as spatial inconsistency, time inconsistency, and food non-standard, will lead to information asymmetry among governments, enterprises, and consumers (21). The high information asymmetry, high externality, high liquidity, and high risk of online food trading (22), as well as the trusted product characteristics of food safety and the self-interest motivation of various stakeholders in the process of food trading, make food safety problems prone to occur in the market (23). However, excessive dispersion of food producers, low market access threshold for food sellers, lack of platform food safety supervision system, and difficulty in tracing the food transportation process will further increase the online food safety problem (24). Among them, the lack of safety awareness of food sellers is the fundamental reason for the existence of online food safety problems (19). The failure of the market reputation mechanism, the imperfect government supervision mechanism, the low threshold for market entry, the imperfect platform information generation and release mechanism, and the opaque platform credit evaluation mechanism are the main reasons for the “lemon problem” in the online food trading market (16, 25).

On the other hand, the governance of online food safety problems. The governance of online food safety problems involves multiple subjects and links, and has many characteristics, such as open structure, complementary functions, subject cooperation, consultation and interaction, and self-regulation. At present, scholars mainly explore the governance of online food safety from the perspective of government, media, online food trading platform, and consumers.

### Government

The government is the direct subject of online food safety governance and plays an important role in the formulation of food safety system (26, 27). The government can alleviate the food safety problem by increasing the inspection probability of enterprises and improving the punishment of self-discipline (28). In relevant research, Ortega et al. found that Chinese consumers have the highest willingness to pay for government certification programs, followed by third-party certification, traceability systems, and product specific information labels (29). A strict monitoring system can not only improve consumers’ welfare, but also restore consumers’ trust and increase social welfare. However, in the online food trading market, there are many and scattered food suppliers, heavy food safety supervision tasks, backward food safety supervision technology, and other problems, which make the defects of the government’s single governance of the online food trading market increasingly prominent (30, 31). Among them, Deng believed that the single government supervision model is a system obstacle to produce food safety risks. It is an inevitable choice for the reform of the food safety supervision model to move from a single government supervision model to a social co governance model (32). Hu pointed out that at present, China’s food safety supervision is faced with such dilemmas as endogenous conflict of policy objectives, structural mismatch of resources and powers, and adverse incentive of regulatory behavior (33). In view of the problems existing in government governance, scholars have given relevant solutions. Among them, Liu and Ma believed that the government should strengthen food safety education to enable the public to have basic knowledge (34). Wei and Yao pointed out that the government should improve the security system of data governance, clarify the operator access mechanism, limit the monopoly of network giants, build a cooperative rights protection mechanism, and reform the regulatory governance system (12). Yang et al. believed that the government should increase punishment, reduce supervision costs, strengthen self-discipline of enterprises, guide the public to participate in governance, and build a multi-agent collaborative governance mechanism (35).

### Media

In order to effectively solve the failure of government regulation and improve the problem of insufficient government regulation, some scholars introduced media to participate in the governance of online food safety problems. Cao et al. studied the role of new media in government food safety supervision and found that efficient and accurate new media supervision can effectively restrain food enterprises’ adulteration behavior and urge the government to perform due diligence supervision (36). Chen et al. believed that the media would take the lead in exposing food safety problems, grasp the guidance of public opinion, and help government regulators to strengthen supervision (37). Zhang et al. found that strengthening third-party supervision is conducive to promoting government regulatory authorities to strengthen supervision and improve enterprise food safety governance (38). Xie et al. found that the sensitivity of producers to perceived reputation loss, the subjective value judgment of media participation in social governance, and the government’s normalization of regulatory penalties are three important constraints for media participation in social governance of food safety (39). Zhang et al. found that reducing the cost of media supervision will not only stimulate the enthusiasm of consumers to complain, but also improve the efficiency of food safety supervision, so that food safety risks are kept at a low level for a long time (40). However, the media have the characteristics of timeliness and low cost and high income, which makes it difficult for them to fully understand the whole process of food events, and it is easy for them to report in a partial way (21, 41).

### Online food trading platform

In the online food trading market, the online food trading platform is the bridge connecting food sellers and food buyers, and the main carrier of online food sales. Compared with the government, food sellers, food buyers, media, and other subjects, online food trading platforms have more advantages in information acquisition, collation, and analysis. They are direct participants in online food safety governance and have the rights and obligations to manage online food trading market (42, 43). Cheng and Dong pointed out that the online food trading platform should give play to its own advantages in technology, information and resources, take the initiative to undertake food seller information review, food information disclosure and other work, so as to ease the regulatory pressure of the government (44). Zhang et al. found that the daily supervision and management of online food trading platform on food safety is crucial to improve the level of food safety supervision on the platform (24). Although the online food trading platform can improve the supervision efficiency of the market, the lack of direct judicial punishment power and the market behavior of pursuing profit maximization will also make the platform “fail” in the process of online food safety governance (45). Therefore, in the online food trading market, it is necessary to strengthen the government’s supervision on the platform and strengthen the platform’s supervision responsibility (21).

### Consumers

With the continuous occurrence of food safety incidents, consumers’ safety awareness has gradually increased and participated in the governance of online food safety problems. Wang and Miao found that consumers’ participation in supervision will affect the production decisions of food enterprises, and enterprises will eventually transform to producing high-quality products (46). Wang and Sha believed that consumers’ education level and objective cognitive ability had a positive impact on online food safety risk prevention and control (47). Niu and Wu believed that the public should be encouraged to supervise and report, and the public interest litigation system and punitive compensation system should be established and improved (48). Zhu and Rong found that the increase of consumers’ real evaluation and complaints about rights protection can effectively promote manufacturers to provide high-quality products (49).

With the deepening of research, scholars found that the single subject regulation could not meet the needs of the rapid development of online food trading market. Therefore, it is very important to integrate the government, media, consumers, platforms, industry associations, and other subjects into the collaborative governance framework of online food safety through legalization, marketization, and other ways, and improve the collaborative governance capability through communication and cooperation (50, 51).

Game theory is a theory that uses rigorous mathematical models to study the optimal decision-making problem under the condition of conflict confrontation (52). In previous studies, Liu et al. built a signaling game model between online food trading platforms and sellers based on signaling game theory, and analyzed the formation conditions and results of different equilibria (53). Zhang et al. constructed a principal-agent model from the perspective of food sellers and food buyers, revealed the equilibrium evolution path of food quality and safety, and proved that the flooding of unsafe food in the market is the inevitable result of non-optimal equilibrium under the asymmetric information environment (23). Evolutionary game theory is a theory that combines game theory analysis with dynamic evolutionary process analysis. This method can help understand the dynamic process of group evolution, and explain why and how groups will reach this state. It has been widely used in management, economics, biology and many other fields (54). For example, in terms of environmental governance, Chu et al. built an evolutionary game model involving the central government, local governments and pollution enterprises, hoping to provide solutions for regional haze governance from the perspective of environmental regulation (55). In terms of online public opinion management, Wen constructed a game model for the evolution of online public opinion in colleges and universities involving the media, college students, universities and the government, and found that the main factors affecting the balance of the game system were the government’s supervision, the willingness of online media to report, the attention of colleges and universities to public opinion events, and college students’ self-awareness (56).

In recent years, scholars have also applied evolutionary game theory to online food safety governance. Liu constructed a static game payment matrix between the government and enterprises, and found that the combination of incentives and government supervision can effectively guide food enterprises to produce safe food (57). Xu et al. established an evolutionary game model involving suppliers and manufacturers, and found that the quality input strategy of food suppliers and manufacturers is closely related to the quality input-output ratio of both parties (58). Zhu and Sun built a tripartite evolutionary game model involving the government, food enterprises, and third-party testing institutions, and analyzed the interaction mechanism of strategy choices among different actors and the evolution trend of each subject’s strategy choices under different parameter changes (59). Wang et al. constructed an evolutionary game model of the behavior of the government and the seller involved in the platform supervision, and analyzed the strategies of the government and the seller under different supervision strengths of the platform (60). Cao et al. built a game model involving the government, the platform and the seller to discuss the collaborative supervision of the government and the platform on online food safety (61).

Therefore, this article constructs a tripartite evolutionary game model with the seller, buyer, and food trading platform, and analyzes the formation process and impact mechanism of the “lemon problem” in the online food trading market. The main contributions of this article are as follows: First, previous studies mainly analyzed online food safety from the theoretical level. However, this article focuses on the “lemon problem” in the online food trading market, and intuitively shows the formation process of the “lemon problem” in the online food trading market from the perspective of dynamic evolution, which can provide new evidence for the existence of the “lemon market.” Second, previous studies mainly focused on the relationship between the government, food sellers, and food trading platforms in the online food trading market, and mostly used the government and food trading platforms to regulate the behavior of food sellers. However, as direct participants in food transactions, food buyers’ behavior also has an important impact on the development and evolution of the market. Therefore, this article mainly analyzes the direct participants in the online food trading market, food sellers, food buyers, and food trading platforms, hoping to have a clearer understanding of the strategic choice of each subject. Third, previous studies mostly analyzed the conditions that affect the subject’s strategic choice behavior, and emphasized the role of supervision, but rarely conducted a comprehensive and in-depth analysis of the factors that affect the subject’s behavior. Therefore, based on the relevant conditions (stability conditions of evolutionary equilibrium) that affect the choice of the subject’s strategy, this article conducts an in-depth analysis of the factors that affect the choice of the subject’s strategy, and clarifies the extent and effect of different factors such as cost, punishment, subsidy, and income on each subject, which can provide a scientific basis for the online food trading platform and the government to formulate relevant institutional systems and policy measures.

## Evolutionary game model in the online food trading market

### Basic assumptions

Without considering the environment of online food trading market, it is assumed that seller, buyer, and online food trading platform can constitute a complete online food trading market based on the functions of each subject in the market. Assuming that each participant is a finite rational individual with information asymmetry among them, the following assumptions are made for the tripartite subjects based on evolutionary game theory.

#### Game subject 1: Seller

With the rise of online food trading, the number of users participating in food transactions has gradually increased, and people’s willingness and ability to pay has also grown, which will greatly promote the benefits of food seller. Under the supervision of the online food trading platform, the food seller will take initiative to provide quality products and services to the buyer in order to maintain user stickiness and attract more buyers. At the same time, with the development of online food trading market, the phenomenon of serious homogenization of products, unclear supply information, and uneven quality levels has become increasingly severe, and platforms problems such as high commissions (the platform will charge the seller a higher sales commission after the sale of products) and overbearing treaties (unfair treaties imposed on the seller and buyer by the platform to escape responsibility and obtain more benefits) have become increasingly prominent, which makes some sellers choose to provide low-quality products to obtain higher profits. Therefore, the seller’s strategy in the online food trading market is {positive performance, negative performance}. Among them, the seller will provide the buyer with high-quality products when choosing “positive performance,” and provide the buyer with low-quality products when choosing “negative performance.” Suppose that the probability of the seller choose “positive performance” is *x* (0 ≤ *x* ≤ 1), the probability of choose “negative performance” is 1 − *x*, where *x* is the function of time *t*, and the initial willingness of the seller is *x*_0_ (0 ≤ *x*_0_ ≤ 1). In the case of the seller choose negative performance, the production cost of food is *C*_1_. In the case of the seller choose positive performance, the seller would put more effort *M* into producing food, and the cost is *M* + *C*_1_. In the case of the buyer choose positive participation, the basic benefit of the seller with positive performance is *R*_1_, and the basic benefit of the seller with negative performance is *R*_2_. In the case of the buyer choose negative participation, the basic benefit of the seller with positive performance is *R*_3_, and the basic benefit of the seller with negative performance is *R*_4_. When the online food trading platform choose “positive regulation,” the seller with positive performance will receive extraneous benefit *R*_5_.

#### Game subject 2: Buyer

In the online food trading market, the seller provides the buyer with a diverse range of products. When buyer faces low-quality products, on the one hand, they will adopt various methods to safeguard their legitimate rights and interests, and on the other hand, they will adopt an indifferent attitude due to process, cost, their own knowledge level, and other reasons. Furthermore, some buyers will release false information for personal benefit, and such speculative behavior will seriously affect the order of the online food trading market. Therefore, the buyer’s strategy in the online food trading market is {positive participation, negative participation}. Among them, the buyer will participate in platform governance and maintain their rights when choosing “positive participation”, and the buyer will not participate in platform governance when choosing “negative participation.” Suppose that the probability of the buyer choose “positive participation” is *y* (0 ≤ *y* ≤ 1), and the probability of choose “negative participation” is 1 − *y*, where *y* is the function of time *t*, and the initial willingness of the buyer is *y*_0_ (0 ≤ *y*_0_ ≤ 1). In the case of the seller choose positive performance, the basic benefit of the buyer is *R*_6_. In the case of the seller choose negative performance, the basic benefit of the buyer with negative participation is *R*_7_, and the basic benefit of the buyer with positive participation is *R*_7_ + *N*. Among them, *N* is the punishment imposed by the buyer with positive participation on the seller with negative performance. When the buyer choose “positive participation,” they need to identify the quality of food and take certain measures to defend their rights, which will require certain cost *C*_2_ . When the online food trading platform choose “positive regulation,” the buyer with positive participation will receive extraneous benefit *R*_8_.

#### Game subject 3: Online food trading platform

In the rapid development process of the online food trading market, the online food trading platform may invest a lot of resources to regulate the seller’s behavior and audit the food quality. Meanwhile, in order to attract more sellers and reduce the operating cost of platform, the online food trading platform may take a laissez-faire attitude toward the speculative behavior of the seller. Therefore, the strategy of the online food trading platform is {positive regulation, negative regulation}. Among them, when the platform choose “positive regulation,” they will manage the behavior of the seller and buyer, while when the platform choose “negative regulation,” they will not respond to the behavior of the seller and buyer. Suppose that the probability of the platform choose “positive regulation” is *z* (0 ≤ *z* ≤ 1), the probability of choose “negative regulation” is 1 − *z*, where *z* is the function of time *t*, and the initial willingness of the platform is *z*_0_ (0 ≤ *z*_0_ ≤ 1). In the case of positive regulation, the online food trading platform will give subsidies *H* to the seller with positive performance, and subsidies *I* to the buyer with positive participation, the platform will give punishment *J* to the seller with negative performance, and punishment *K* to the buyer with negative participation. When the online food trading platform choose “positive regulation,” they will supervise the behavior of seller and buyer, which will incur certain regulation cost *C*_3_. In addition, when the seller choose “positive performance,” the platform will receive the perceived benefit *R*_9_ due to the improvement of reputation, user scale and brand value. And when the buyer choose “positive participation,” the food trading platform will receive the perceived benefit *R*_10_.

**FIGURE 1:**
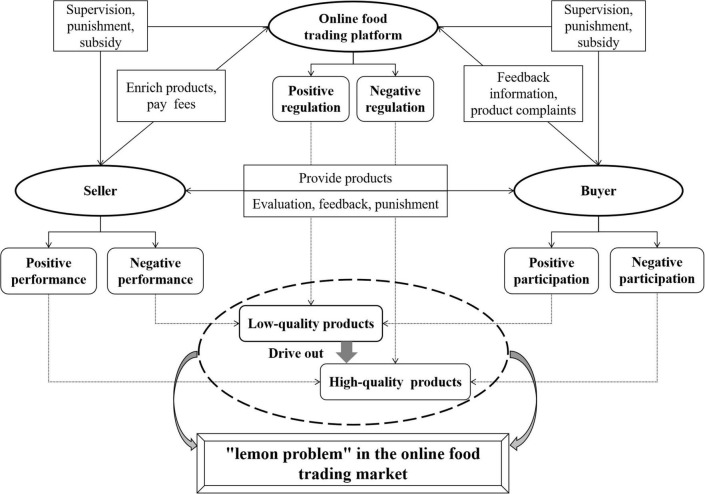
Formation process of the “lemon problem” in the online food trading market.

In the online food trading market, when food sellers provide low-quality products, buyers passively protect their rights, and online food trading platforms allow food sellers to speculate, there will be more and more low-quality food sellers in the market, and promote a large number of high-quality food sellers to leave the market. At this time, the phenomenon of “bad money drives out good money” will appear in the online food trading market, which is also known as the “lemon problem.” The formation process of the “lemon problem” in the online food trading market is shown in Figure 1, the relevant parameters and their meanings are shown in Table 1.

**TABLE 1:** Symbols and meanings of parameters.

Symbols	Description
*M*	Cost difference between positive performance and negative performance of the seller
*N*	Punishment from the buyer with positive participation to the seller with negative performance
*H*	Subsidy from the platform with positive regulation to the seller with positive performance
*I*	Subsidy from the platform with positive regulation to the buyer with positive participation
*J*	Punishment from the platform with positive regulation to the seller with negative performance
*K*	Punishment from the platform with positive regulation to the buyer with negative participation
*R*_1_	Basic benefit of the seller with positive performance when the buyer choose positive participation
*R*_2_	Basic benefit of the seller with negative performance when the buyer choose positive participation
*R*_3_	Basic benefit of the seller with positive performance when the buyer choose positive participation
*R*_4_	Basic benefit of the seller with negative performance when the buyer choose negative participation
*R*_5_	Extraneous benefit of the seller with positive performance when the platform choose positive regulation
*R*_6_	Basic benefit of the buyer when the seller choose positive performance
*R*_7_	Basic benefit of the buyer with negative participation when the seller choose negative performance
*R*_8_	Extraneous benefit of the buyer with positive participation when the platform choose positive regulation
*R*_9_	Perceived benefit of the platform when the seller choose positive performance
*R*_10_	Perceived benefit of the platform when the buyer choose positive participation
*C*_1_	Input cost when the seller choose negative performance
*C*_2_	Input cost when the buyer choose positive participation
*C*_3_	Input cost when the platform choose positive regulation
*x*	Probability of the seller choose positive performance
*y*	Probability of the buyer choose positive participation
*z*	Probability of the platform choose positive regulation

### Construction of tripartite game model

Based on the above analysis assumptions, a benefit matrix of the tripartite game model is constructed with the seller, the buyer and the online food trading platform as the tripartite subjects. The benefits of all subjects under different scenarios are shown in Table 2. Among them, when the strategy of the seller, buyer and platform is {positive performance, positive participation, positive regulation}, the seller’s benefit is *R*_1_ + *R*_5_ + *H* − *M* − *C*_1_, the buyer’s benefit is *R*_6_ + *R*_8_ + *I* − *C*_2_, and the platform’s benefit is *R*_9_ + *R*_10_ − *H* − *I* − *C*_3_.

**TABLE 2:** Benefit matrix of the tripartite game of seller, buyer, and platform.

Seller	Buyer	Online food trading platform	
		Positive regulation_*z*_	Negative regulation_1–z_
Positive	Positive	*R*_1_ + *R*_5_ + *H* − *M* − *C*_1_	*R*_1_ − *M* − *C*_1_
performance *x*	participation	*R*_6_ + *R*_8_ + *I* − *C*_2_	*R*_6_ − *C*_2_
	_*y*_	*R*_9_ + *R*_10_ − *H* − *I* − *C*_3_	*R*_9_ + *R*_10_
	Negative	*R*_3_ + *R*_5_ + *H* − *M* − *C*_1_	*R*_3_ − *M* − *C*_1_
	participation	*R*_6_ − *K*	*R*_6_
	1 − *y*	*R*_9_ + *K* − *H* − *C*_3_	*R*_9_
Negative	Positive	*R*_2_ − *N* − *J* − *C*_1_	*R*_2_ − *N* − *C*_1_
performance	participation	*R*_7_ + *R*_8_ + *N* + *I* − *C*_2_	*R*_7_ + *N* − *C*_2_
1 − *x*	*y*	*R*_10_ + *J* − *I* − *C*_3_	*R*_10_
	Negative	*R*_4_ − *J* − *C*_1_	*R*_4_ − *C*_1_
	participation	*R*_7_ − *K*	*R*_7_
	1 − *y*	*J* + *K* − *C*_3_	0

When the seller, buyer and platform choose different strategies, they will get different benefits, as shown below.

#### Seller

The expected benefit when the seller choose “positive performance” is:


$$E_{x}=y\left(R_{1}-R_{3}\right)+z\left(R_{5}+H\right)+R_{3}-M-C_{1}$$

The expected benefit when the seller choose “negative performance” is:


$$E_{1-x}=y\left(R_{2}-N-R_{4}\right)-zJ+R_{4}-C_{1}$$

The average expected benefit of the seller is:


$$\bar{E_{a}}=xE_{x}+\left(1-x\right)E_{1-x}$$

The replicator dynamic equation of the seller is:


(1) $$\begin{array}{l} U(x)=\frac{dx}{dt}=x(E_{x}-\bar{E_{a}})=x(1-x)[y(R_{1}+R_{4}-R_{2}-R_{3}\\ \;+N)+z(R_{5}+H+J)+R_{3}-R_{4}-M]. \end{array}$$

#### Buyer

The expected benefit when the buyer choose “positive participation” is:


$$E_{y}=x\left(R_{6}-R_{7}-N\right)+z\left(R_{8}+I\right)+R_{7}+N-C_{2}$$

The expected benefit when the buyer choose “negative participation” is:


$$E_{1-y}=x\left(R_{6}-R_{7}\right)-zK+R_{7}$$

The average expected benefit of the buyer is:


$$\bar{E_{b}}=yE_{y}+\left(1-y\right)E_{1-y}$$

The replicator dynamic equation of the buyer is:


(2) $$\begin{array}{l} U(y)=\frac{dy}{dt}=y(E_{y}-\bar{E_{b}})=y(1-y)[z(R_{8}+I+K)\\ \;+N(1-x)-C_{2}]. \end{array}$$

#### Online food trading platform

The expected benefit when the online food trading platform choose “positive regulation” is:


$$E_{z}=x\left(R_{9}-H-J\right)+y\left(R_{10}-I-K\right)+J+K-C_{4}$$

The expected benefit when the online food trading platform choose “negative regulation” is:


$$E_{1-z}=xR_{9}+yR_{10}$$

The average expected benefit of the online food trading platform is:


$$\bar{E_{c}}=zE_{z}+\left(1-z\right)E_{1-z}$$

The replicator dynamic equation of the online food trading platform is:


(3) $$\begin{array}{l} U(z)=\frac{dz}{dt}=z(E_{z}-\bar{E_{c}})=z(1-z)[x(-H-J)+y(-I-K)\\ \;+J+K-C_{3}]. \end{array}$$

### Stability analysis

By combining Formulae 1–3, the replicator dynamic system of the online food trading market can be obtained.


(4) $$\left\{ \begin{array}{ccc} U\left(x\right)=x\left(1-x\right)[y\left(R_{1}+R_{4}-R_{2}-R_{3}+N\right) & & \\ \;+z\left(R_{5}+H+J\right)+R_{3}-R_{4}-M] & & \\ U\left(y\right)=y\left(1-y\right)\left[z\left(R_{8}+I+K\right)+N\left(1-x\right)-C_{2}\right] & & \\ U\left(z\right)=z\left(1-z\right)\left[x\left(-H-J\right)+y\left(-I-K\right)+J+K-C_{3}\right] & & \end{array}\right.$$

When *U*(*x*) = 0 ⋂ *U*(*y*) = 0 ⋂ *U*(*z*) = 0 in Formula 4, the equilibrium points of the replicator dynamic system can be obtained: *E*_*1*_(0,0,0), *E*_2_(0,0,1), *E*_3_(0,1,0), *E*_4_(0,1,1), *E*_5_(1,0,0), *E*_6_(1,0,1), *E*_7_(1,1,0), *E*_8_(1,1,1), and *E*_9_(x*, y*, z*). In Su et al. (62), Xiao et al. (63), and other studies, scholars believe that the Evolutionary Stable Strategy (ESS) of the multi group evolutionary game must also be a pure strategy Nash equilibrium, that is, in an asymmetric game, the mixed strategy equilibrium must not be an evolutionary stability equilibrium. Therefore, this article will only analyze eight pure strategy equilibrium points, that is, do not discuss *E*_*9*_(x*, y*, z*). The stability of each equilibrium point in the tripartite evolutionary game can be determined according to the Lyapunov stability theory, that is, when the replicator dynamic system is evolutionary stable, the eigenvalue of its Jacobian matrix is negative (64).

By calculating the partial derivatives of *U*(*x*), *U*(*y*) and *U*(*z*) for *x, y*, and *z*, respectively, the Jacobian matrix of replicator dynamic system of online food trading market can be obtained. The eigenvalues can be calculated by substituting the values of *x, y*, and *z* at each equilibrium point into Formula 5. For example, the eigenvalues of *E*_1_ (0,0,0) is λ_1_ = *R*_3_ − *R*_4_ − *M*, λ_2_ = *N* − *C*_2_, λ_3_ = *J* + *K* − *C*_3_. The eigenvalues of each equilibrium point are shown in Table 3.

**TABLE 3:** Eigenvalues of equilibrium point.

Equilibrium point	λ_1_	λ_2_	λ_3_
*E*_1_ (0,0,0)	*R*_3_ − *R*_4_ − *M*	*N* − *C*_2_	*J* + *K* − *C*_3_
*E*_2_ (0,0,1)	*R*_5_ + *H* + *J* + *R*_3_ − *R*_4_ − *M*	*R*_8_ + *I* + *K* + *N* − *C*_2_	− (*J* + *K* − *C*_3_)
*E*_3_ (0,1,0)	*R*_1_ + *N* − *R*_2_ − *M*	− (*N* − *C*_2_)	*J* − *I* − *C*_3_
*E*_4_ (0,1,1)	*R*_1_ + *R*_5_ − *R*_2_ + *N* + *H* + *J* − *M*	− (*R*_8_ + *I* + *K* + *N* − *C*_2_)	− (*J* − *I* − *C*_3_)
*E*_5_ (1,0,0)	− (*R*_3_ − *R*_4_ − *M*)	− *C*_2_	*K* − *H* − *C*_3_
*E*_6_ (1,0,1)	− (*R*_5_ + *H* + *J* + *R*_3_ − *R*_4_ − *M*)	*R*_8_ + *I* + *K* − *C*_2_	− (*K* − *H* − *C*_3_)
*E*_7_ (1,1,0)	− (*R*_1_ + *N* − *R*_2_ − *M*)	*C*_2_	− *H* − *I* − *C*_3_
*E*_8_ (1,1,1)	− (*R*_1_ + *R*_5_ − *R*_2_ + *N* + *H* + *J* − *M*)	− (*R*_8_ + *I* + *K* − *C*_2_)	*H* + *I* + *C*_3_


(5) $$J=[\begin{array}{cc} \left(1-2x\right)\left[\begin{array}{c} y\left(R_{1}+R_{4}-R_{2}-R_{3}+N\right)+\\ z\left(R_{5}+H+J\right)+R_{3}-R_{4}-M \end{array}\right]\; & \\ -y\left(1-y\right)N\; & \\ z\left(1-z\right)\left(-H-J\right)\; & \end{array}$$

$$\begin{array}{cc} x\left(1-x\right)\left(R_{1}+R_{4}-R_{2}-R_{3}+N\right)\; & \\ \left(1-2y\right)\left[\begin{array}{c} z\left(R_{8}+I+K\right)+\\ N\left(1-x\right)-C_{2} \end{array}\right]\; & \\ z\left(1-z\right)\left(-I-K\right)\; & \end{array}$$

$$\begin{array}{cc} x\left(1-x\right)\left(R_{5}+H+J\right)\; & \\ y\left(1-y\right)\left(R_{8}+I+K\right)\; & \\ \left(1-2z\right)\left[\begin{array}{c} x\left(-H-J\right)+y(-I\\ -K)+J+K-C{}_{3} \end{array}\right]\; & \end{array}]$$

For each equilibrium point, if its eigenvalues λ_1_, λ_2_, λ_3_ are all negative, then it is the evolutionary stable point of the system. According to the actual operation of the online food trading market, *C*_2_ > 0 and *H* + *I* + *C*_3_ > 0 can be known by analyzing the equilibrium points. That is to say, the two equilibrium points *E*_7_ (1,1,0) and *E*_8_ (1,1,1) are unstable. If it is to achieve the evolution stability, the asymptotical stability of equilibrium points *E*_1_ (0,0,0), *E*_2_ (0,0,1), *E*_3_ (0,1,0), *E*_4_ (0,1,1), *E*_5_ (1,0,0), and *E*_6_ (1,0,1) need to be analyzed. The asymptotical stability conditions for each equilibrium point are shown in Table 4.

**TABLE 4:** Asymptotical stability conditions for the replicator dynamic system at equilibrium points.

Equilibrium point	Asymptotical stability conditions	Number
*E*_1_ (0,0,0)	*R*_3_ − *R*_4_ − *M* < 0, *N* − *C*_2_ < 0, *J* + *K* − *C*_3_ < 0	➀
*E*_2_ (0,0,1)	*R*_5_ + *H* + *J* + *R*_3_ − *R*_4_ − *M* < 0, *R*_8_ + *I* + *K* + *N* − *C*_2_ < 0, − (*J* + *K* − *C*_3_) < 0	➁
*E*_3_ (0,1,0)	*R*_1_ + *N* − *R*_2_ − *M* < 0, − (*N* − *C*_2_) < 0, *J* − *I* − *C*_3_ < 0	➂
*E*_4_ (0,1,1)	*R*_1_ + *R*_5_ − *R*_2_ + *N* + *H* + *J* − *M* < 0, − (*R*_8_ + *I* + *K* + *N* − *C*_2_) < 0, − (*J* − *I* − *C*_3_) < 0	➃
*E*_5_ (1,0,0)	− (*R*_3_ − *R*_4_ − *M*) < 0, − *C*_2_ < 0, *K* − *H* − *C*_3_ < 0	➄
*E*_6_ (1,0,1)	− (*R*_5_ + *H* + *J* + *R*_3_ − *R*_4_ − *M*) < 0, *R*_8_ + *I* + *K* − *C*_2_ < 0, − (*K* − *H* − *C*_3_) < 0	➅

According to Table 4, we can know that many factors, such as the seller’s benefit, the seller’s cost, the buyer’s cost, the platform’s cost, the platform’s subsidy and punishment to the seller, the platform’s subsidy and punishment to the buyer, and the buyer’s punishment to the seller, will affect the eigenvalues of each equilibrium point. The stability of each equilibrium point in the asymptotic stability conditions ➀–➅ will be analyzed below (Table 5).

**TABLE 5:** Stability of each equilibrium point under each asymptotic stability condition.

		*E*_1_	*E*_2_	*E*_3_	*E*_4_	*E*_5_	*E*_6_
➀	Plus or minus	—	?? +	? + −	?? +	+ –	?? +
	Stability	ESS	Unstable	Unstable	Unstable	Unstable	Unstable
➁	Plus or minus	– +	—	? + ?	? + ?	+ − ?	+ − ?
	Stability	Unstable	ESS	Unstable	Unstable	Unstable	Unstable
➂	Plus or minus	? + ?	? + ?	—	? − +	? − ?	???
	Stability	Unstable	Unstable	ESS	Unstable	Uncertain	Uncertain
➃	Plus or minus	?? +	? + ?	− ? +	—	? − ?	???
	Stability	Unstable	Unstable	Unstable	ESS	Uncertain	Uncertain
➄	Plus or minus	+ ??	+ ??	???	???	—	− ? +
	Stability	Unstable	Unstable	Unstable	Uncertain	ESS	Unstable
➅	Plus or minus	?? +	+ ??	???	???	? − +	—
	Stability	Unstable	Unstable	Uncertain	Uncertain	Unstable	ESS

According to Table 5, under each asymptotic stability condition, there may be multiple equilibrium points in the replicator dynamic system. Specific conditions are as follows:

In the case of condition ➀, except that *E*_1_ is the stable point of the Jacobian matrix of the replicator dynamic system, other equilibrium points are all unstable points. At this time, {negative performance, negative participation, negative regulation} is the evolutionary stability point of the system.

In the case of condition ➁, there is only *E*_2_ as a stable point in the system. At this time, {negative performance, negative participation, positive regulation} is the evolutionary stability point of the system.

In the case of condition ➂, *E*_3_ is the stable point, that is, {negative performance, active participation, negative regulation} is the evolutionary stable point of the system. In addition, the stability of *E*_5_ and *E*_6_ is uncertain. The specific conditions are as follows: When conditions ➂ and ➄ are met simultaneously, *E*_3_ and *E*_5_ are stable points and *E*_6_ is unstable point. When conditions ➂ and ➅ are met simultaneously, *E*_3_ and *E*_6_ are stable points and *E*_5_ is unstable point. In general, under condition ➂, there are at most two stable points in the system.

In the case of condition ➃, *E*_4_ is the stable point, and the stability of *E*_5_ and *E*_6_ is uncertain. The specific conditions are as follows: When conditions ➃ and ➄ are met simultaneously, *E*_4_ and *E*_5_ are stable points and *E*_6_ is unstable point. When conditions ➃ and ➅ are met simultaneously, *E*_4_ and *E*_6_ are stable points and *E*_5_ is unstable point. In general, under condition ➃, there are at most two stable points in the system.

In the case of condition ➄, *E*_5_ is the stable point, and the stability of *E*_3_ and *E*_4_ is uncertain. The specific conditions are as follows: When conditions ➄ and ➂ are met simultaneously, *E*_5_ and *E*_3_ are stable points and *E*_4_ is unstable point. When conditions ➄ and ➃ are met simultaneously, *E*_5_ and *E*_4_ are stable points and *E*_3_ is unstable point. In general, under condition ➄, there are at most two stable points in the system.

In the case of condition ➅, *E*_6_ is the stable point, and the stability of *E*_3_ and *E*_4_ is uncertain. The specific conditions are as follows: When conditions ➅ and ➂ are met simultaneously, *E*_6_ and *E*_3_ are stable points and *E*_4_ is unstable point. When conditions ➅ and ➃ are met simultaneously, *E*_6_ and *E*_4_ are stable points and *E*_3_ is unstable point. In general, under condition ➅, there are at most two stable points in the system.

By analyzing the stability of the equilibrium points in the asymptotical stability conditions ➀–➅, it can be found that there may be multiple stable points in the replicator dynamic system under various asymptotic stability conditions. Specifically, when condition ➀ or condition ➁ is met, there will be only one stable point in the system. When condition ➂ and ➄, ➂ and ➅, ➃ and ➄, ➃ and ➅ are met respectively, there will be two stable points in the system.

## The formation and evolution of the “lemon problem” in the online food trading market

### Parameter setting

For different sellers, buyers and online food trading platforms, the initial strategic choice behavior may be affected in many ways and show some differences. In order to avoid this impact, this article refers to the method of setting the initial value of the system in the previous evolutionary game analysis (65), and sets the initial willingness of each subject to low, medium, and high levels, that is _*x_0_,y_0_,z_0_*_ ∈ Ω(0.2, 0.5, 0.8). By combining the initial willingness of the three subjects, 27 different scenarios can be obtained. For the convenience of comparison, the following only explores the cases where the initial willingness of each subject are consistent.

In the process of assigning values to each variable, first, we set the variable parameters according to the stability of condition ➀, that is, each variable needs to meet *R*_3_ − *R*_4_ − *M* < 0, *N* − *C*_2_ < 0, *J* + *K* − *C*_3_ < 0 at the same time. After constant debugging, we finally determined the parameters of condition ➀. Then, for the convenience of exploring the changing situation of each subject in the online food trading market, this article takes the condition ➀ as the base, with reference to the actual operating structure and interest relationship of the online food trading market, and assign values for variables ➀, ➁, ➂, ➃, ➄, ➅, ➂ and ➄, ➂ and ➅, ➃ and ➄, and ➃ and ➅for different situations. For example, for condition ➁, it is difficult to satisfy *R*_5_ + *H* + *J* + *R*_3_ − *R*_4_ − *M* < 0, *R*_8_ + *I* + *K* + *N* − *C*_2_ < 0, − (*J* + *K* − *C*_3_) < 0 by substituting the variable parameters in condition ➀. To this end, it is necessary to compare the difference between condition ➀ and condition ➁, and adjust on the basis of the relevant parameters of condition ➀. It is found by comparison that: condition ➁ can be met when *M, C*_2_, and *C*_3_ are adjusted, condition ➂ can be met when *M* and *N* are adjusted, condition ➃ can be met when *M* and *J* are adjusted, condition ➄ can be met when *R*_3_ is adjusted, condition ➅ can be met when *K* and *C*_2_ are adjusted, condition ➂ and ➄ can be met when *M, N, C*_2_, and *R*_3_ are adjusted, condition ➂ and ➅ can be met when *M, N, K, C*_2_, and *R*_3_ are adjusted, condition ➃and ➅ can be met when *M, J*, and *R*_3_ are adjusted, condition ➂ and ➅ can be met when *M, N, J, K, C*_2_, and *R*_3_ are adjusted. The specific assignment of each variable under different conditions is shown in Table 6.

**TABLE 6:** Variable assignment in the online food trading market.

Conditions	*M*	*N*	*H*	*I*	*J*	*K*	*C*_2_	*C*_3_	*R*_1_	*R*_2_	*R*_3_	*R*_4_	*R*_5_	*R*_8_
➀	2	1	1	1	1	1	2	3	22	21	2	1	1	1
➁	5	1	1	1	1	1	5	1	22	21	2	1	1	1
➂	5	3	1	1	1	1	2	3	22	21	2	1	1	1
➃	10	1	1	1	5	1	2	3	22	21	2	1	1	1
➄	2	1	1	1	1	1	2	3	22	21	4	1	1	1
➅	2	1	1	1	1	5	8	3	22	21	2	1	1	1
➂ and ➄	4	2	1	1	1	1	1	3	22	21	6	1	1	1
➂ and ➅	11	9	1	1	1	5	8	3	22	21	10	1	1	1
➃ and ➄	10	1	1	1	5	1	2	3	22	21	12	1	1	1
➃ and ➅	11	2	1	1	5	5	8	3	22	21	6	1	1	1

### Simulation analysis

In the online food trading market, many conditions will occur if the system is to reach evolutionary stable equilibrium. The following simulates and analyzes the strategic choice behaviors of each subject under different conditions.

#### Simulation analysis under asymptotical stability condition ➀

**FIGURE 2:**
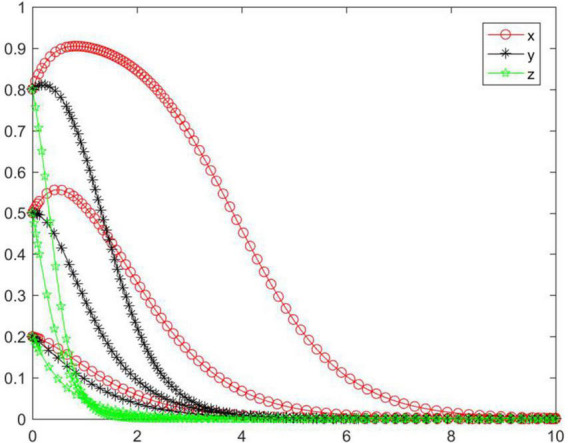
Tripartite strategy evolution trends under condition ➀.

In the case of condition ➀, the strategy of the tripartite subjects will eventually stabilize in {negative performance, negative participation, negative regulation} (Figure 2). In the case of condition ➀, the strategy tend of platform will rapidly reach a negative state, while the seller and buyer will reach a negative state relatively slowly. As the initial willingness increases, the speed and probability of each subject evolving to a negative state gradually decrease. With medium and high initial willingness, the seller’s strategy will first evolve to a positive state and then to a negative state, and the higher the initial willingness, the more obvious the seller’s evolving trend will be toward a positive state. Combined with condition ➀, we can know that *M, N, J, K, C*_2_, *C*_3_, and *R*_4_ will have an impact on the strategic choice behavior of all subjects, and the initial willingness will promote all subjects to choose positive strategy to a certain extent.

#### Simulation analysis under asymptotical stability condition ➁

**FIGURE 3:**
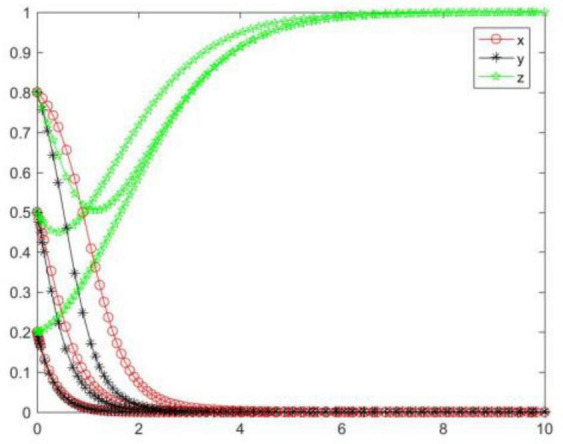
Tripartite strategy evolution trends under condition ➁.

In the case of condition ➁, the strategy of the tripartite subjects will eventually stabilize in {negative performance, negative participation, positive regulation} (Figure 3). In the case of condition ➁, the strategy of the seller and buyer will rapidly evolve to a negative state, and the lower the initial willingness, the greater the speed and probability of the subject evolving to a negative state. For the online food trading platform, its strategy will evolve to a negative state first and then to a positive state, and the higher the initial willingness, the more obvious the trend of the platform evolving toward a negative state. Combining conditions ➀ and ➁, we can know that *M, N, H, I, J, K, C*_2_, *C*_3_, *R*_3_, and *R*_4_ will have an impact on the strategic choice behavior of all subjects, and the change of *M, C*_2_, and *C*_3_ will promote the platform’s strategy to stabilize in a positive state.

#### Simulation analysis under asymptotical stability condition ➂

**FIGURE 4:**
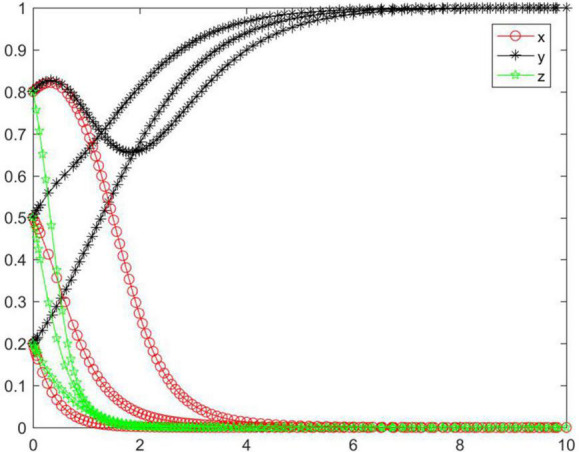
Tripartite strategy evolution trends under condition ➂.

In the case of condition ➂, the strategy of the tripartite subjects will eventually stabilize in {negative performance, positive participation, negative regulation} (Figure 4). In the case of condition ➂, the strategy of the platform will rapidly evolve to a negative state, and the lower the initial willingness, the greater the speed and probability of the subject evolving to a negative state. The seller evolves relatively slowly toward a negative state. In the case with high initial willingness, the seller also has a tendency to evolve to a positive state. The buyer’s strategy will evolve toward a positive state. In the case with medium initial willingness, the speed and probability of the buyer evolving to a positive state are greater, followed by low initial willingness. And with high initial willingness, the buyer is the slowest to evolve to a positive state, and its strategy will fluctuate to a certain degree in the early period. Combining conditions ➀ and ➂, we can know that *M, N, I, J, K, C*_2_, *C*_3_, *R*_1_, and *R*_2_ will have an impact on the strategic choice behavior of all subjects, and the change of *M* and *N* will promote the buyer’s strategy to stabilize in a positive state.

#### Simulation analysis under asymptotical stability condition ➃

**FIGURE 5:**
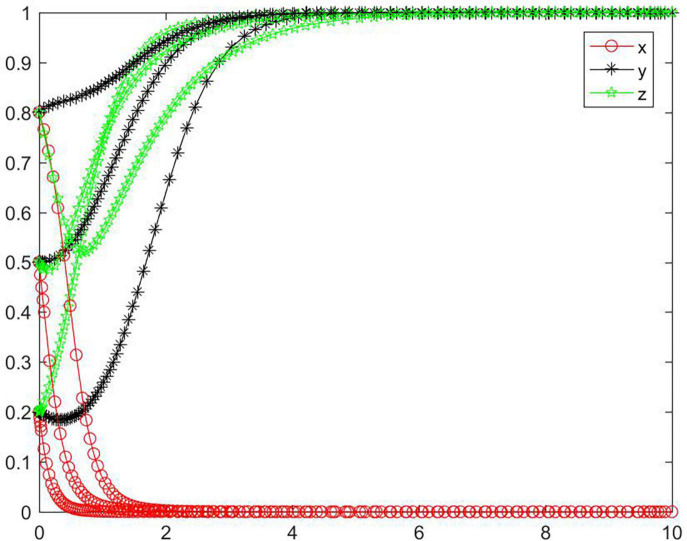
Tripartite strategy evolution trends under condition ➃.

In the case of condition➃, the strategy of the tripartite subjects will eventually stabilize in {negative performance, positive participation, positive regulation} (Figure 5). In the case of condition ➃, the seller’s strategy will quickly evolve to a negative state, and the lower the initial willingness, the greater the speed and probability that the subject tends to reach a negative state. The strategy of the buyer and platform gradually evolve to a positive state. With increasing initial willingness, the speed and probability of the buyer evolving to a positive state gradually increase, while the speed and probability of the platform evolving to a positive state gradually decrease. Combining conditions ➀ and ➃, we can know that *M, N, H, I, J, K, C*_2_, *C*_3_, *R*_1_, *R*_2_, *R*_5_, and *R*_8_ will have an impact on the strategic choice behavior of all subjects, and the change of *M* and *J* can promote the strategy of the buyer and platform to be stable in a positive state.

#### Simulation analysis under asymptotical stability condition ➄

**FIGURE 6:**
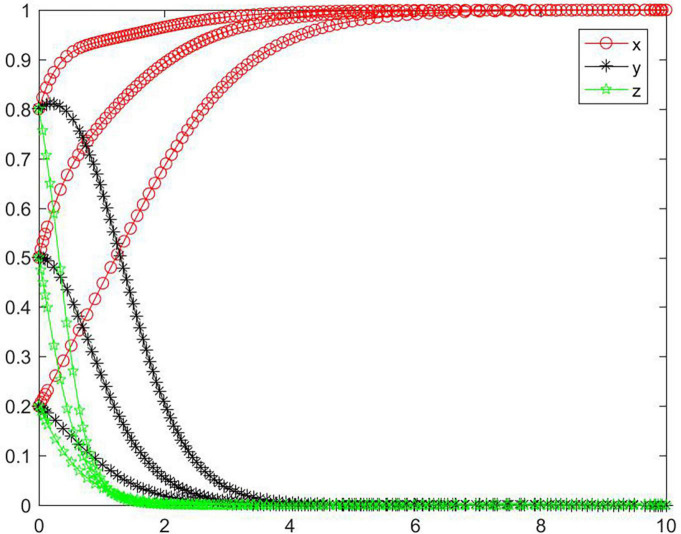
Tripartite strategy evolution trends under condition ➄.

In the case of condition ➄, the strategy of the tripartite subjects will eventually stabilize in {positive performance, negative participation, negative regulation} (Figure 6). In the case of condition ➄, the strategy of the buyer and platform gradually evolves to a negative state, and the lower the initial willingness, the higher the speed and probability of the subject evolving toward a negative state. The seller’s strategy gradually evolves to a positive state, and the higher the initial willingness, the faster and more likely the subject evolves to a positive state. Combining conditions ➀ and ➄, we can know that *M, H, K, C*_2_, *C*_3_, *R*_3_, and *R*_4_ will have an impact on the strategic choice behavior of all subjects, and the change of *R*_3_ will promote the seller’s strategy to stabilize in a positive state.

#### Simulation analysis under asymptotical stability condition ➅

**FIGURE 7:**
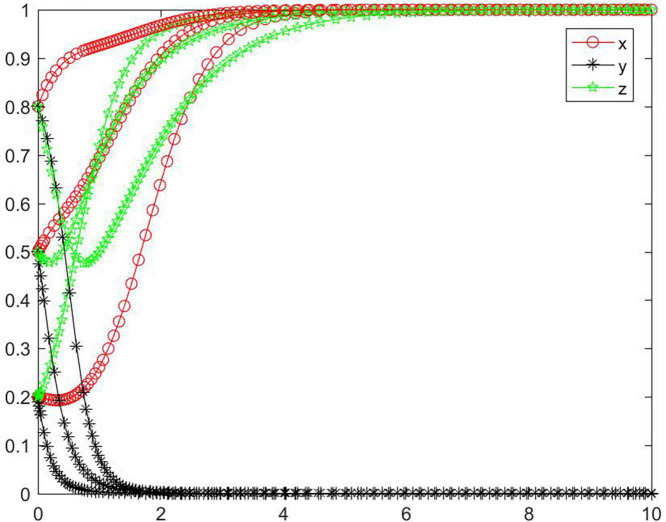
Tripartite strategy evolution trends under condition ➅.

In the case of condition ➅, the strategy of the tripartite subjects will eventually stabilize in {positive performance, negative participation, positive regulation} (Figure 7). In the case of condition ➅, the buyer’s strategy quickly evolves to a negative state, and the lower the initial willingness, the greater the speed and probability of the subject evolving toward a negative state. The strategy of the seller and the platform gradually evolve to a positive state. With the increase of initial willingness, the speed and probability of the seller’s strategy evolving to a positive state gradually increase, while the speed and probability of the online food trading platform evolving to a positive state gradually decrease. Combining conditions ➀ and ➅, we can know that *M, H, I, J, K, C*_2_, *C*_3_, *R*_3_, *R*_4_, *R*_5_, and *R*_8_ will have an impact on the strategic choice behavior of all subjects, and the change of *K* and *C*_2_ can promote the strategy of the seller and the platform to be stable in a positive state.

#### Simulation analysis under asymptotical stability conditions ➂ and ➄

**FIGURE 8:**
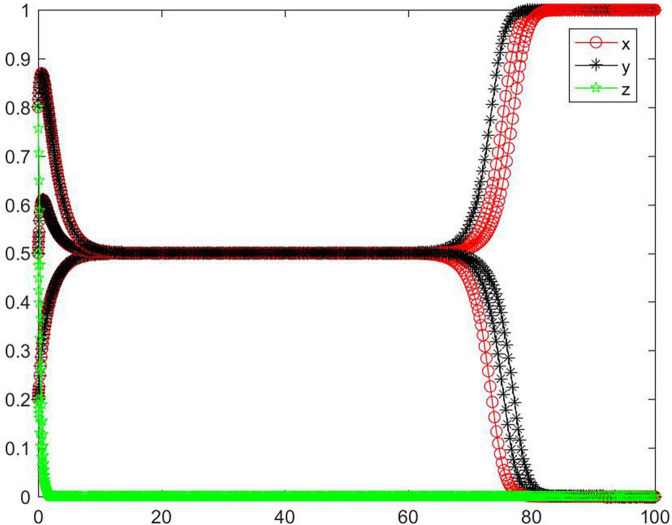
Tripartite strategy evolution trends under conditions ➂ and ➄.

In the cases of conditions ➂ and ➄, the strategy of the tripartite subjects will eventually stabilize in {negative performance, positive participation, negative regulation} and {positive performance, negative participation, negative regulation} (Figure 8). It can be seen that, in the cases of conditions ➂ and ➄, the strategy of the online food trading platform rapidly evolves to a negative state, and the lower the initial willingness, the higher the speed and probability of the subject evolving toward a negative state. The strategy of the seller and the buyer diverge as the system evolves. In the case of low and high initial willingness, the seller’s strategy is eventually positive, and the buyer’s strategy is negative; when the initial willingness is medium, the seller’s strategy evolves to be at a negative state, and the buyer’s strategy evolves to a positive state. Combining conditions ➀ and ➂, ➄, we can know that *M, N, H, I, J, K, C*_2_, *C*_3_, *R*_1_, *R*_2_, *R*_3_, *R*_4_ will have an impact on the strategic choice behavior of all subjects, and the change of *M, N, C*_2_, and *R*_3_ can promote the strategy of the seller and the buyer to be stable in different states.

#### Simulation analysis under asymptotical stability conditions ➂ and ➅

**FIGURE 9:**
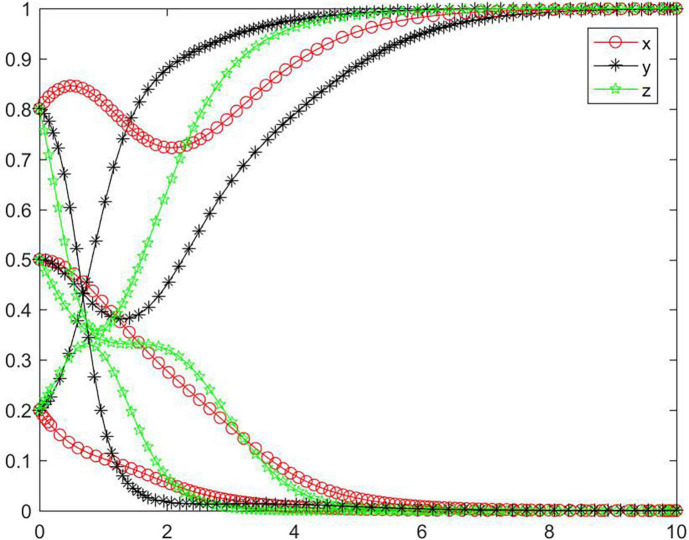
Tripartite strategy evolution trends under conditions ➂ and ➅.

In the cases of conditions ➂ and ➅, the strategy of the tripartite subjects will eventually stabilize in {negative performance, positive participation, negative regulation} and {positive performance, negative participation, positive regulation} (Figure 9). When the initial willingness is low and medium, the strategy of the seller and the platform evolve to a negative state, and the buyer’s strategy evolves to a positive state. When the initial willingness is high, the strategy of the seller and the online food trading platform evolves to a positive state, and the buyer’s strategy evolves to a negative state. Combining conditions ➀ and ➂, ➅, we can know that *M, N, H, I, J, K, C*_2_, *C*_3_, *R*_1_, *R*_2_, *R*_3_, *R*_4_, *R*_5_, and *R*_8_ will have an impact on the strategic choice behavior of all subjects, and the change of *M, N, K, C*_2_, and *R*_3_ can promote the strategy of seller, buyer and platform to be stable in different states.

#### Simulation analysis under asymptotical stability conditions ➃ and ➄

**FIGURE 10:**
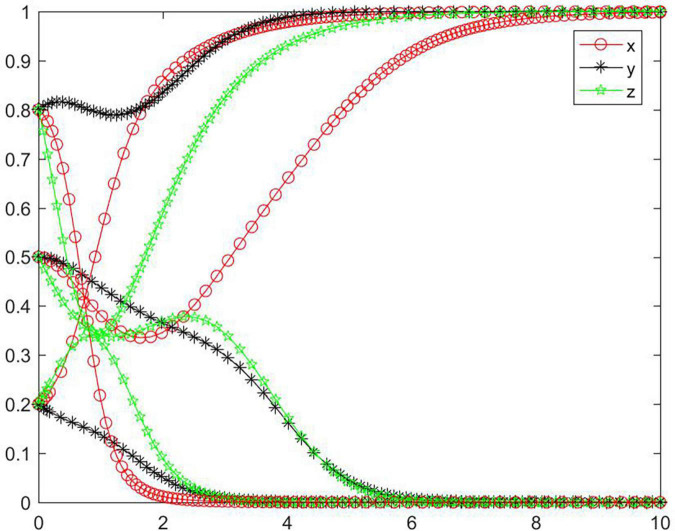
Tripartite strategy evolution trends under conditions ➃ and ➄.

In the cases of conditions ➃ and ➄, the strategy of the tripartite subjects will eventually stabilize in {negative performance, positive participation, positive regulation} and {positive performance, negative participation, negative regulation} (Figure 10). When the initial willingness is low and medium, the strategy of the seller evolve to a positive state, and the strategy of the buyer and the platform evolve to a negative state. When the initial willingness is high, the strategy of the buyer and the platform evolve to a positive state, and the seller’s strategy evolves to a negative state. Combining conditions ➀ and ➃, ➄, we can know that *M, N, H, I, J, K, C*_2_, *C*_3_, *R*_1_, *R*_2_, *R*_3_, *R*_4_, *R*_5_, and*R*_8_ will have an impact on the strategic choice behavior of all subjects, and the change of *M, J*, and *R*_3_ can promote the strategy of seller, buyer and platform to be stable in different states.

#### Simulation analysis under asymptotical stability conditions ➃ and ➅

**FIGURE 11:**
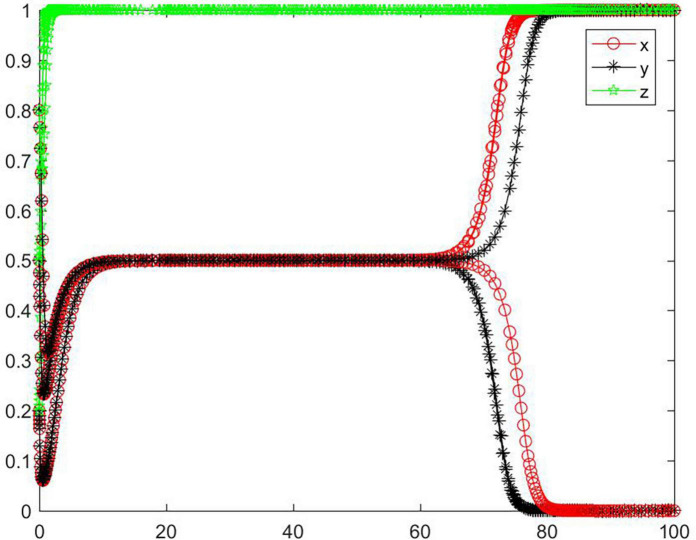
Tripartite strategy evolution trends under conditions ➃ and ➅.

In the cases of conditions ➃ and ➅, the strategy of the tripartite subjects will eventually stabilize in {negative performance, positive participation, positive regulation} and {positive performance, negative participation, positive regulation} (Figure 11). When the initial willingness is low and medium, the strategy of the seller and the platform evolve to a positive state, and the buyer’s strategy evolves to a negative state. When the initial willingness is high, the strategy of the buyer and the online food trading platform evolve to a positive state, and the seller’s strategy evolves to a negative state. Combining conditions ➀ and ➃, ➅, we can know that *M, N, H, I, J, K, C*_2_, *C*_3_, *R*_1_, *R*_2_, *R*_3_, *R*_4_, *R*_5_, and *R*_8_ will have an impact on the strategic choice behavior of all subjects, and the change of *M, N, J, K, C*_2_, and *R*_3_ can promote the strategy of the platform to be stable in a positive state, and promote the strategy of seller and buyer to be stable in different states.

### Analysis of the formation mechanism of the “lemon problem” in the online food trading market

Under different conditions, the strategy of seller, buyer, and platform in the online food trading market will eventually stabilize in six situations.

In the cases of {negative performance, negative participation, negative regulation}, {negative performance, negative participation, positive regulation}, {negative performance, positive participation, negative regulation}, {negative performance, positive participation, positive regulation}, the seller will eventually choose negative strategy. That is to say, with the development of the online food trading market, more and more sellers on the market will choose to provide low-quality products, and the seller who choose to provide high-quality products will gradually be crowded out of the market, eventually leading to the “lemon problem” in the online food trading market.

In the case of {positive performance, negative participation, negative regulation}, the seller will eventually choose positive strategy, the buyer and the platform will choose negative strategy. Although the seller will choose positive strategy in this case, satisfying this scenario requires that the benefits the seller adopts positive strategy obtains are far greater than those of the seller adopting negative strategy. In actual situations, when the buyer and the online food trading platform choose negative strategy, the seller may obtain higher benefits from providing low-quality products.

In the case of {positive performance, negative participation, positive regulation}, the seller and the platform will choose positive strategy, while the buyer will choose negative strategy. However, in order to meet this scenario, it will be more costly for the buyer to positively participate, and the platform will also have to impose severer punishment on negative buyer. In reality, the cost of for buyer with positive participation is relatively low, and the platform is more to encourage buyer to choose negative performance than to impose higher punishment on the buyer. Because high punishment will cause a large number of buyer withdraw from the market, and make the entire online food trading market disappear.

In general, during the development and evolution of the online food trading market, the “lemon problem” will occur and will not disappear. To solve the “lemon problem” in the online food trading market, it is necessary to analyze the factors affecting the development and evolution of the online food trading market and formulate the corresponding measures to suppress and reduce the occurrence of the “lemon problem.”

## Influencing factors analysis of the “lemon problem” in the online food trading market

Through the analysis of asymptotical stability conditions ➀–➅, it is found that the strategy of each subject in the online food trading market will be affected by variables like *M, N, H, I, J, K, C*_2_, *C*_3_, *R*_1_, *R*_2_, *R*_3_, *R*_4_, *R*_5_, and *R*_8_. In the above research, we only analyzed the changes of each subject’s strategic behavior under the common changes of multiple factors. In order to analyze the strategy choice behavior of each subject in depth and find the core elements that affect the strategy change of each subject, this article makes specific analyses of the factors on the basis of asymptotical stability condition ➀.

### Cost of positive strategy

**FIGURE 12:**
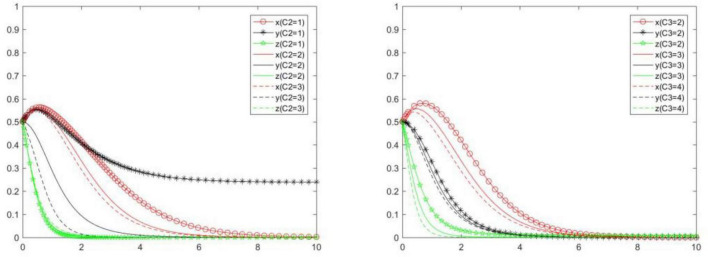
Tripartite strategy evolution trends when *C*_2_ and *C*_3_ change.

In order to explore the influence of *C*_2_ and *C*_3_ on the choice of each subject’s strategy, we will analyze the evolution law of each subject’s strategy under scenarios {*C*_2_ = 1, *C*_2_ = 2, *C*_2_ = 3}, {*C*_3_ = 2, *C*_3_ = 3, *C*_3_ = 4}. Compare and analyze the evolution law of each subject’s strategy when *C*_2_ and *C*_3_ change (Figure 12). We can know the following.

When *C*_2_ changes, the strategies of the seller and buyer will change significantly, and the platform will change little. Specifically, with the increase of *C*_2_, the probability and speed of the seller and buyer to choose positive strategy will gradually decrease, and the probability and speed of the platform to choose positive strategy will gradually increase. In other words, *C*_2_ will have a negative impact on the positive strategy choice of the seller and the buyer, and will have a positive impact on the positive strategy choice of the online food trading platform. The reason is that if a large amount of investment cannot be exchanged for the same or more benefits, the buyer’s enthusiasm for participating in market governance and rights protection will also be greatly reduced. The reduction of the buyer’s willingness to defend their rights will reduce the supervision of the seller’s behavior and make them tend to choose negative strategy. At this time, in order to maintain market order and retain more users, the food trading platform will choose positive strategy.

When *C*_3_ changes, the strategies of the seller, buyer, and platform will change to some extent. Specifically, with the increase of *C*_3_, the probability and speed of the seller, buyer, and platform to choose positive strategy will gradually decrease. In other words, *C*_3_ will have a negative impact on the positive strategy choice of the seller, buyer, and platform. The reason is that when the platform increases the regulation cost, it will restrict the behavior of the seller and buyer from many aspects, and will transfer the cost to the seller and buyer, which will reduce the enthusiasm of the seller and buyer and drive them to withdraw from the online food trading market.

### Punishment of positive strategy

**FIGURE 13:**
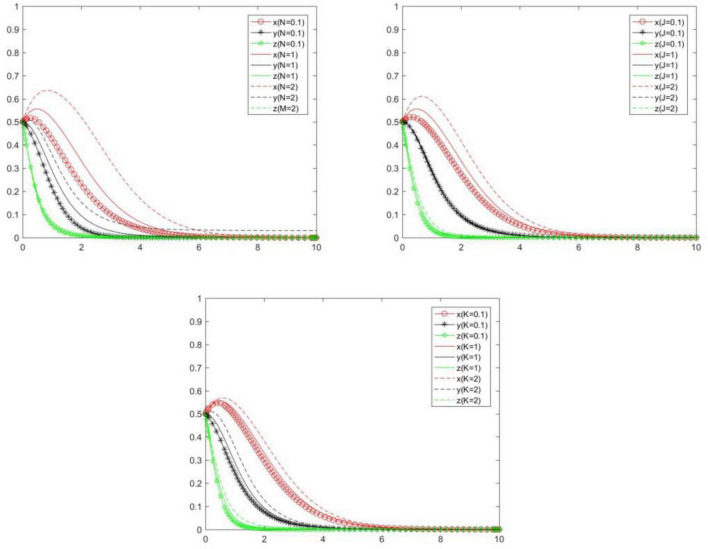
Tripartite strategy evolution trends when *N, J*, and *K* change.

In order to explore the influence of *N, J*, and *K* on the choice of each subject’s strategy, we will analyze the evolution law each subject’s strategy under scenarios {*N* = 0.1, *N* = 1, *N* = 2}, {*J* = 0.1, *J* = 1, *J* = 2}, {*K* = 0.1, *K* = 1, *K* = 2}. Compare and analyze the evolution law of each subject’s strategy when *N, J*, and *K* change (Figure 13). We can know the following.

When *N* changes, the strategies of the seller and buyer will change significantly, and platform will change slightly. Specifically, with the increase of *N*, the probability and speed of the seller and buyer to choose positive strategy will gradually increase, and the probability and speed of the platform to choose positive strategy will gradually decrease. In other words, *N* will have a positive impact on the positive strategy choice of the seller and buyer, and will have a negative impact on the positive strategy choice of the online food trading platform.

When *J* changes, the seller will change significantly, the online food trading platform will change slightly, and the buyer will not change. Specifically, with the increase of *J*, the probability and speed of the seller and platform to choose positive strategy will gradually increase. In other words, *J* will have a positive impact on the positive strategy choice of the seller and platform.

When *K* changes, the seller, the buyer, and the online food trading platform will change to some extent. Specifically, with the increase of *K*, the probability and speed of the seller, buyer, and platform to choose positive strategy will gradually increase. In other words, *K* will have a positive impact on the positive strategy choice of the seller, buyer and platform.

In general, a certain degree of punishment will prompt relevant subjects to choose positive strategy. Because the benefits obtained in the process of punishment will urge the subject who implements punishment to choose positive strategy, while the subject who accepts punishment will also choose positive strategy in order to avoid losses.

### Subsidy and extraneous benefit of positive strategy

**FIGURE 14:**
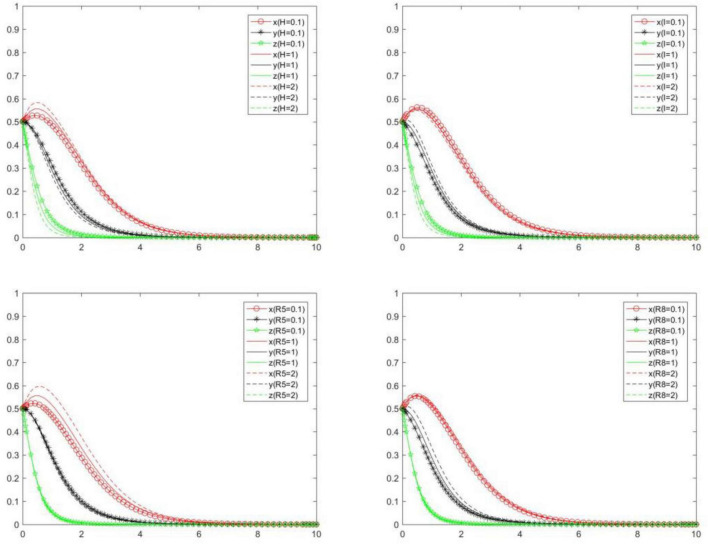
Tripartite strategy evolution trends when *H, I, R5*, and *R8* change.

In order to explore the influence of *H, I, R*_5_, and *R*_8_ on the choice of each subject’s strategy, we will analyze the evolution law of each subject’s strategy under scenarios {*H* = 0.1, *H* = 1, *H* = 2}, {*I* = 0.1, *I* = 1, *I* = 2}, {*R*_5_ = 0.1, *R*_5_ = 1, *R*_5_ = 2}, {*R*_8_ = 0.1, *R*_8_ = 1, *R*_8_ = 2}. Compare and analyze the evolution law of each subject’s strategy when *H, I, R*_5_ and *R*_8_ change (Figure 14). We can know the following.

When *H, I, R*_5_, and *R*_8_ change, the strategies of the seller, buyer and platform will change slightly. This is mainly because the decision on how much subsidy and additional benefit the subject can obtain is made by other subjects rather than itself. From the perspective of interests, other entities will not provide them with more subsidies, which will not make major changes due to changes in *H, I, R*_5_, and *R*_8_.

With the increase of *H*, the probability and speed of the seller to choose a positive strategy will gradually increase, while the probability and speed of the buyer and platform to choose a positive strategy will gradually decrease. That is to say, *H* will have a positive impact on the positive strategy choice of the seller, and will have a negative impact on the positive strategy choice of the buyer and the online food trading platform.

With the increase of *I*, the probability and speed of the seller and platform to choose a positive strategy will gradually decrease, while the probability and speed of the buyers to choose a positive strategy will gradually increase. That is to say, *I* will have a negative impact on the positive strategy choice of the seller and platform, and will have a positive impact on the positive strategy choice of the buyer.

With the increase of *R*_5_, the probability and speed of the seller to choose a positive strategy will gradually increase, while the probability and speed of the buyer and platform to choose a positive strategy will gradually decrease. That is to say, *R*_5_ will have a positive impact on the positive strategy choice of the seller, and will have a negative impact on the positive strategy choice of the buyer and platform.

With the increase of *R*_8_, the probability and speed of the seller and buyer to choose a positive strategy will gradually increase, while the probability and speed of the platform to choose a positive strategy will gradually decrease. That is to say, *R*_8_ will have a positive impact on the positive strategy choice of the seller and buyer, and will have a negative impact on the positive strategy choice of the platform.

### Cost and benefit difference between positive strategy and negative strategy

**FIGURE 15:**
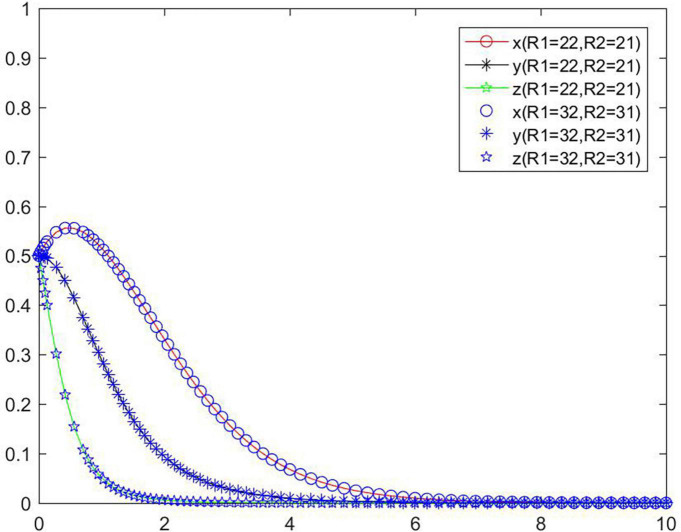
Tripartite strategy evolution trends when *R1* and *R*_2_ change and *R*_1_ − *R*_2_ unchanged.

**FIGURE 16:**
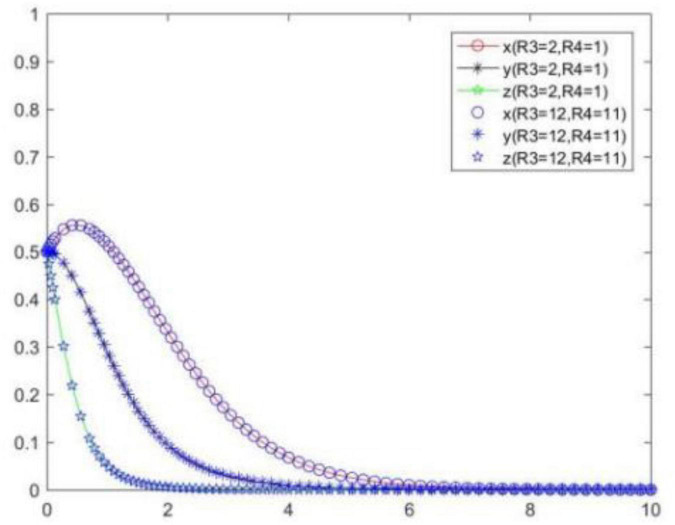
Tripartite strategy evolution trends when *R*_3_ and *R*_4_ change and *R*_3_ − *R*_4_ unchanged.

The analysis shows that *R*_1_, *R*_2_, *R*_3_, and *R*_4_ do not directly affect the evolution game of the system, but affect the system through the benefit difference between the positive and negative performance of the seller, that is, affect the evolution law of the system through *R*_1_ − *R*_2_ and *R*_3_ − *R*_4_. Figure 15 shows the tripartite strategy evolution trends when *R*_1_ and *R*_2_ change and *R*_1_ − *R*_2_ unchanged, and Figure 16 shows the tripartite strategy evolution trends when *R*_3_ and *R*_4_ change and *R*_3_ − *R*_4_ unchanged.

**FIGURE 17:**
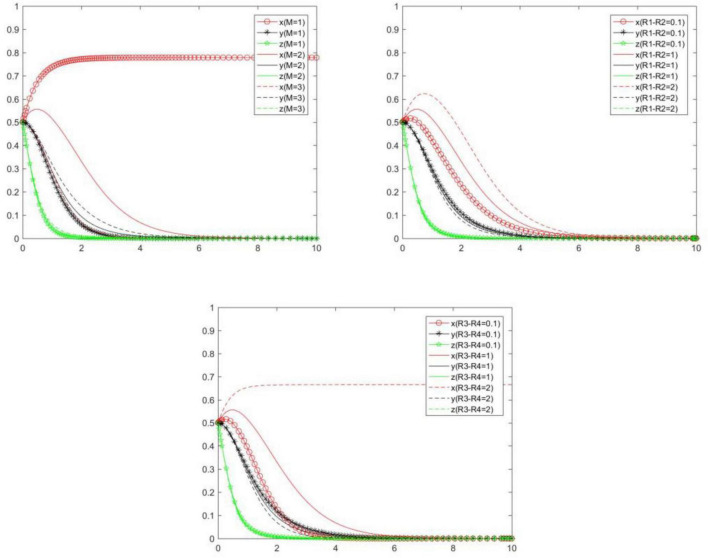
Tripartite strategy evolution trends when *M, R*_1_ − *R*_2_, and *R*_3_ − *R*_4_ change.

In order to explore the influence of *M, R*_1_ − *R*_2_, and *R*_3_ − *R*_4_ on the choice of each subject’s strategy, we will analyze the evolution law of each subject’s strategy under scenarios {*M* = 1, *M* = 2, *M* = 3}, {*R*_1_ − *R*_2_ = 0.1, *R*_1_ − *R*_2_ = 1, *R*_1_ − *R*_2_ = 2}, {*R*_3_ − *R*_4_ = 0.1, *R*_3_ − *R*_4_ = 1, *R*_3_ − *R*_4_ = 2}. Compare and analyze the evolution law of each subject’s strategy when *M, R*_1_ − *R*_2_, and *R*_3_ − *R*_4_ change (Figure 17). We can know the following.

When *M, R*_1_ − *R*_2_, and *R*_3_ − *R*_4_ change, the seller’s strategy changes most significantly, followed by the buyer, and the platform’s strategy changes less. The reason is that when the cost difference and benefit difference between the positive and negative performance of the seller are large, the seller will quickly react and choose strategies that are beneficial to itself, and the seller’s different strategies will also have an impact on the buyer and cause them to change indirectly.

With the increase of *M*, the probability and speed of the seller to choose a positive strategy will gradually decrease, and the probability and speed of the buyer and platform to choose a positive strategy will gradually increase. That is to say, *M* will have a negative impact on the positive strategy choice of the seller, and will have a positive impact on the positive strategy choice of the buyer and platform.

With the increase of *R*_1_ − *R*_2_ and *R*_3_ − *R*_4_, the probability and speed of the seller to choose a positive strategy will gradually increase, and the probability and speed of the buyer and platform to choose a positive strategy will gradually decrease. That is to say, *R*_1_ − *R*_2_ and *R*_3_ − *R*_4_ will have a positive impact on the positive strategy choice of the seller, and will have a negative impact on the positive strategy choice of the buyer and platform.

## Conclusion and policy implications

### Conclusion

Through systematic analysis of the structure and operation mode of the online food trading market, this article takes the seller, buyer, and online food trading platform as tripartite subjects and constructs a tripartite evolution game model. With the help of MATLAB software, it is possible not only to simulate the formation process of the “lemon problem” in the online food trading market dynamically, but also to conduct an in-depth analysis of the strategic choice behavior of each subject. In general, the following conclusions can be drawn.

Through the analysis of the stable points of evolution of the seller, buyer, and online food trading platform, it is found that the “lemon problem” occurs with the development and evolution of the online food trading market and will not dissipate. In the online food trading market, the strategy of the tripartite subjects will stabilize in six situations. And the “lemon problem” occurs in all six situations. In the research of Zhang et al., it was also pointed out that under the network trading environment of asymmetric information, the characteristics of trusted products of food safety and the self-interest motives of various stakeholders in the food supply chain make unsafe food flooding the market an inevitable result (23). In order to control the “lemon problem” in the online food trading market, it is necessary to deeply analyze the factors that affect the development and evolution of the market, and use advanced technical means and effective management mechanisms to alleviate the problem of information asymmetry in the market (66, 67).

In the online food trading market, different factors have different influence directions on the subject strategy. For the seller, *N, H, J, K, R*_1_ − *R*_2_, *R*_3_ − *R*_4_, *R*_*5*_, and *R*_*8*_ will have a positive impact on the seller’s positive strategy choice, while *M, I, C*_*2*_, and *C*_*3*_ will have a negative impact on the seller’s positive strategy choice. For the buyer, *M, N, I, K*, and *R*_*8*_ will have a positive impact on the buyer’s positive strategy choice, while *H, C*_*2*_, *C*_*3*_, *R*_1_ − *R*_2_, *R*_3_ − *R*_4_, and *R*_*5*_ will have a negative impact on the buyer’s positive strategy choice. For the online food trading platform, *M*, J, *K*, and *C*_*2*_ will have a positive impact on the platform’s positive strategy choice, while *N, H, I, C*_*3*_, *R*_1_ − *R*_2_, *R*_3_ − *R*_4_, *R*_*5*_, and *R*_*8*_ will have a negative impact on the platform’s positive strategy choice. Clarifying the impact of different factors on each subject can not only help understand the strategic choice behavior of each subject in the online food trading market, but also help and guide the subject’s behavior from various aspects. For example, we can change *M, J, K*, and *C*_*2*_ to promote the platform to choose a positive strategy.

In the online food trading market, different factors have different influence degrees on the subject strategy. Among them, *M, N, H, I, J, K, C*_2_, *C*_3_, *R*_1_ − *R*_2_, *R*_3_ − *R*_4_, and *R*_5_ will have a significant impact on the seller’s strategic choice behavior, *M, N, K, C*_2_, *C*_3_, and *R*_8_ will have a significant impact on the buyer’s strategic choice behavior, *H* and *C*_3_ will have a significant impact on the platform’s strategic choice behavior. Analyzing the degree of influence of different factors on the subject’s strategy can help us clearly identify the key factors that affect the subject’s strategy choice and point out the direction for the subsequent development of solutions. However, the degree of influence mentioned in this article is more the result of the comparison among multiple subjects, rather than quantitative comparison. In order to more clearly identify the differences between the impact degrees, we will collect realistic data from various aspects in the future to verify and deepen relevant conclusions.

In the online food trading market, cost, punishment, subsidy and benefit have different effects on the subject’s strategy. Among them, cost and cost difference have the most significant impact on the subject’s strategy, followed by punishment and benefit difference, and subsidy and additional benefit have less impact on the subject’s strategy. Therefore, if we want to build a good and sustainable online food trading market, we need to focus on cost reduction and reveal food quality and safety information at the lowest management cost (68). At present, establishing a good institution (such as signal detection mechanism, reputation mechanism, and reward and punishment mechanism) has become an inevitable requirement for the healthy development of online food trading market (53). However, most of the supervision in the market is ex-post supervision, and although the incentive forms are diverse, there is no clear and scientific basis, which requires comprehensive management of the online food trading market from various aspects.

### Policy implications

In order to effectively suppress the “lemon problem” in the online food trading market, provide consumers with rich and diverse, healthy and safe food, and bring more benefits to food suppliers and food trading platforms, this paper presents the following suggestions.

(1) In order to reduce the input cost of tripartite subjects and improve the quality of information in the market, the online food trading market should establish a complete and standardized examination and verification institution. In the online food trading market, the performance cost of the seller, the participation cost of the buyer, and the regulation cost of the online food trading platform will have influence on the strategic choices of the subjects. On the one hand, a complete examination and verification institution will effectively reduce the cost of information screening for the buyer and the online food trading platform and will encourage the buyer and the platform to adopt positive strategy. On the other hand, it will increase the input cost of the low-quality seller, reduce the competitive pressure of the high-quality seller, and further encourage the seller to adopt positive strategy. Specific improvements can be made in the following aspects.
➀ The quality of information should be strictly controlled from the food source and a complete food seller qualification examining and verifying mechanism should be established. In order to have a general examination of user credibility, creation ability, and consumption ability of product, the online food trading platform can connect user information with bank credit information system, higher education information network, enterprise credit information query platform, etc.
➁ The online food trading market should refine the sale rules of products and standardize the product examining and verifying process to raise the threshold for products entering the market. For example, for certain products, the platform may require the food seller to provide information such as the creation time, place, raw materials, technology, and process of food production, which can not only ensure the safety of products, but also provide a evidence for subsequent accountability and verification.
➂ The online food trading market should improve public rules for examining and verifying reports. The online food trading market can release regulatory information and examine and verify information to users in its reports. Well-designed examining and verifying reports and information releasing rules can improve accountability efficiency, promote platform information transparency, and awe dishonest users.
(2) In order to encourage and restrict the behaviors of the subjects, the online food trading market should establish an appropriate and flexible reward and punishment institution. In the online food trading market, the subsidy received by the seller with positive performance, the punishment for the seller with negative performance, the subsidy received by the buyer with positive participation, and the punishment for the buyer with negative participation will not only affect each subject’s own strategic choice, but will also affect the behavior of other subjects. Generally speaking, subsidy institution can encourage the positive strategic behavior of the subject, and the punishment institution can restrain the negative strategic behavior of the subject. The establishment of an appropriate and flexible reward and punishment institution can not only regulate the behavior of the subjects but also ensure the orderly operation of the online food trading market at the same time. Specific improvements can be made in the following aspects.
➀ Based on the credit file of the seller, the online food trading platform should establish a complete institution integrating signal recognition, detection, and processing, so that the platform can accurately determine the information transacted by the seller. At the same time, the credit records of the food seller also need to be made, which can provide a basis for the platform to set specific standards for reward and punishment.
➁ To make a clear distinction between reward and punishment, the online food trading platform should raise the reward for high-quality food sellers and the punishment for low-quality food sellers. At the same time, the platform should also improve its accuracy and efficiency in detecting information from the seller. For the food seller who have been providing high-quality products for a long time, the online food trading platform can reduce the proportion of their trading commission fee, reduce their advertising and bidding rank fees. For the dishonest food seller, the platform will charge a certain amount of punishment fees and restrict some of their behaviors when the platform detects that he is selling low-quality products at high prices. For the food seller with fraudulent behaviors and food quality problems, the platform may disclose this illegal operation information to all users and remind consumers to be cautious when buying.
➂ The online food trading market can constantly innovate its reward and punishment institution, try to introduce institutions such as expert identification, user reward reporting, media reward monitoring, guide the seller to operate in good faith, and then construct an honest, high-quality, and professional online food trading market environment.
(3) In order to effectively reduce the supervision cost of each subject and improve the supervision efficiency, the online food trading market should establish a supervision institution involving multi-party participation. In the online food trading market, the buyer and online food trading platform will supervise the behavior of seller, and the online food trading platform will supervise the seller and buyer. However, affected by the differences in the user’s knowledge level, the subjectivity of product evaluation and the externalities of product, although all subjects will invest more time and energy to identify products, it is often difficult to achieve the expected supervision goals. To effectively solve the lemon problem in the online food trading market, it is necessary to continuously update the supervision methods and supervision concepts, and actively introduce different supervision subjects to participate in the management (11, 69–71). Specific improvements can be made in the following aspects.
➀ The public and platform should be encouraged to participate in supervision. On the one hand, it is necessary to introduce the online food trading platform to participate in the supervision, give full play to the initiative of each platform, continuously improve the supervision awareness and supervision technology of each platform, so as to build a supervision institution that includes access rules, transaction rules, evaluation rules, etc. On the other hand, the public supervision function should be given full play, a variety of complaint channels can be established to encourage the public to complain and provide suggestions to the supervision institution.
➁ It is necessary to clarify the government’s supervisory responsibilities. In the online food trading market, government participation in supervision can not only more effectively regulate the behavior of the platform and the seller, but also provide more security for food quality and safety (60, 72–74). In the process of supervision, the government should not only have different supervision models and supervision content according to the actual situation, but also actively guide and encourage other subjects to participate in supervision. At the same time, the government should also pay attention to the standards of legislation and law enforcement, not to over-regulate and over-restrict the development of the online food trading market.
➂ Industry associations and internal supervision institutions should be established. In the online food trading market, leading enterprises in the food industry and well-known food bloggers (experts in the food field) can take the lead in establishing industry associations with other users, and promote the self-discipline management of the online food trading market through industry associations. The industry associations can take advantage of the regulatory power, information acquisition and professional technology to internally discuss multiple topics such as market access thresholds, marketing methods, product and service pricing, capital management, and information disclosure, and share the discussion results with the online food trading platform and government (25, 75).

## Data availability statement

The original contributions presented in this study are included in the article/supplementary material, further inquiries can be directed to the corresponding author.

## Author contributions

FS and SF: conceptualization. FS and JC: methodology, software, and writing—original draft preparation. XZ: validation. FS: formal analysis, resources, data curation, and funding acquisition. JC: investigation. FS, XZ, SF, and SA: writing—review and editing. All authors read and agreed to the published version of the manuscript.

## Footnotes

1 The lemons problem refers to concerns concerning the value of an investment or product that develop as a result of unequal knowledge possessed by the buyer and supplier.
